# Mitochondrial disorders leading to Alzheimer’s disease—perspectives of diagnosis and treatment

**DOI:** 10.1007/s11357-024-01118-y

**Published:** 2024-03-08

**Authors:** Magdalena Pszczołowska, Kamil Walczak, Weronika Miśków, Magdalena Mroziak, Justyna Chojdak-Łukasiewicz, Jerzy Leszek

**Affiliations:** 1 Faculty of Medicine, Wrocław Medical University, Wrocław, Poland; 2 Clinic of Psychiatry, Department of Psychiatry, Medical Department, Wrocław Medical University, Wrocław, Poland; 3 Department of Neurology, Wrocław Medical University, Wrocław, Poland

**Keywords:** Mitochondrial dysfunction, Alzheimer’s disease, Oxidative stress, ROS, Antioxidants, MitoQ

## Abstract

Alzheimer’s disease (AD) is a neurodegenerative disorder and the most common cause of dementia globally. The pathogenesis of AD remains still unclear. The three main features of AD are extracellular deposits of amyloid beta (Aβ) plaque, accumulation of abnormal formation hyper-phosphorylated tau protein, and neuronal loss. Mitochondrial impairment plays an important role in the pathogenesis of AD. There are problems with decreased activity of multiple complexes, disturbed mitochondrial fusion, and fission or formation of reactive oxygen species (ROS). Moreover, mitochondrial transport is impaired in AD. Mouse models in many research show disruptions in anterograde and retrograde transport. Both mitochondrial transportation and network impairment have a huge impact on synapse loss and, as a result, cognitive impairment. One of the very serious problems in AD is also disruption of insulin signaling which impairs mitochondrial Aβ removal.

Discovering precise mechanisms leading to AD enables us to find new treatment possibilities. Recent studies indicate the positive influence of metformin or antioxidants such as MitoQ, SS-31, SkQ, MitoApo, MitoTEMPO, and MitoVitE on mitochondrial functioning and hence prevent cognitive decline. Impairments in mitochondrial fission may be treated with mitochondrial division inhibitor-1 or ceramide.

**Graphical Abstract:**

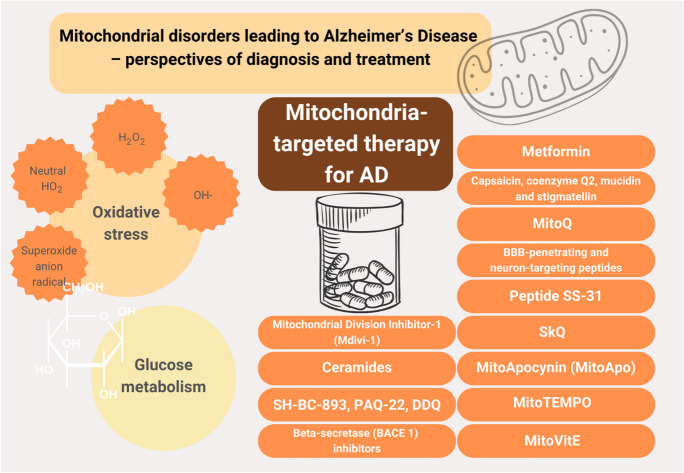

(Graphic content via Canva Pro)

## Introduction

Alzheimer’s disease (AD) is a progressive neurodegenerative disorder characterized by cognitive impairment and mental symptoms. AD is responsible for up to 80% of dementia diagnoses [1]. In 2020, about 44 million people worldwide suffered from AD and this is estimated to double by 2050 [2]. Most AD patients exhibit non-hereditary, late-onset Alzheimer’s disease (LOAD) which is sporadic. Only 5% of all cases of AD are a result of dominant mutations of APP, PSEN1, and PSEN2 genes. The risk of AD increases with older age and genetic factors, such as the ApoE gene, which occurs in 16% of the population. Also, lifestyle and dietary factors increase the probability of Alzheimer’s disease [3–5]. Pathological criteria for AD diagnosis include increased levels of Aβ peptide and hyperphosphorylated tau (*p*-tau) [5]. β-amyloid forms structures called plaques and tau forms neurofibrillary tangles. Both of the proteins are indicators of AD as they are responsible for cell death resulting in loss of brain tissue [6]. The pathogenesis of AD is complex and multifactorial. Although the mechanism of AD pathogenesis is still unclear, the growing evidence suggests that damaged mitochondria play a fundamental role in AD pathogenesis [7]. Several mitochondrial alterations are found in AD [1]. The mitochondrial dysfunction affects amyloid precursor protein (APP) production, APP cleavage, and Aβ accumulation. Moreover, mitochondrial dysfunction can stimulate other molecular changes associated with AD, such as impaired tau phosphorylation and inflammation [7].

## 2. Mitochondrial impairment in AD

Mitochondria are the cell’s energy source, providing most of the ATP through oxidative phosphorylation (OXPHOS). Of all cell types, neurons have one of the highest rates of ATP utilization, which is used primarily to maintain the ionic gradients required for continuous electrophysiological activity, neurotransmission, and short-term synaptic plasticity [8]. Mitochondria involve energy production, communication between brain cells, and detoxification. They regulate temperature and maintain redox balance in cells [9]. Mitochondrial dysfunction causes many pathological cellular processes, such as the production of reactive oxygen species (ROS) and associated oxidative stress, as well as intracellular calcium deregulation and apoptosis. In Alzheimer’s disease, decreased activity of multiple complexes such as complex I (NADH ubiquinone oxidoreductase), complex IV (cytochrome oxidase (COX), and complex V (ATPase) [10] which cooperate in ATP producing process [11], pyruvate dehydrogenase complex, and α-ketoglutarate dehydrogenase complex is observed. In comparison to the age-matched non-AD brains also, activity of phosphofructokinase (PFK), phosphoglycerate mutase, aldolase, glucose-6-phosphate isomerase, and lactate dehydrogenase are reduced. On the other hand, the activity and number of reactive oxygen species (ROS) increased. In the brains of transgenic mice that overexpress human APP, translocase progressively accumulates mitochondrial Aβ, it is connected with reduced activity of ETC complexes III and IV, and the rate of oxygen consumption decreases [10]. The results of the studies revealed that mitochondrial impairment occurs before plaque deposition and, what is more, Aβ-associated mitochondria were observed even 4 months before extracellular Aβ deposits. AD mitochondria not only are small and fragmented but also, as mouse models have shown, have impaired function of mitochondrial fusion and fission [10]]. Fusion and fission are necessary to maintain mitochondrial homeostasis. Imbalance in this process leads to mitochondrial swelling, depolarisation, and fragmentation, increases susceptibility to many forms of neuronal stress, and, as a result, leads to cell death [11]. Mitochondrial trafficking also is impaired by Aβ [10]. Rat model studies have shown that the addition of Aβ to the isolated mitochondria causes mitochondrial dysfunction such as mitochondrial membrane depolarization, ATP/ADP ratio decrease, cytochrome c expulsion, and activation of caspase-3 can be a trigger to the apoptotic cascade [12]. Also, Aβ makes connections between mitochondria and endoplasmic reticulum abnormally tight and impairs Ca2 + signaling, autophagy, motility, and apoptosis. Recently it was shown that the mitochondrial large-conductance Ca2 + -activated potassium channels are directly blocked by Aβ [2]. Ca2 + efflux is also blocked by tau which inhibits mitochondrial Na + /Ca2 + exchanger. Mitochondria are not only energy producers, but also calcium signaling, an important element in cell death. Blocking the Na + /Ca2 + exchanger leads to calcium overload, opening the mitochondrial permeability transition pore (mPTP), and as a result induces cell death [13]. Moreover, the experimental models of AD showed a dysfunction of mitochondrial transport. Mitochondria moves through neurons by kinesin, toward the nerve terminal (anterograde direction), and dynein, toward the soma (retrograde direction). Intracellular movement is necessary to maintain neuronal polarity, neurotransmission, and membrane potential. In AD even before Aβ and tau aggregation, axonal degeneration, as a result of excessive accumulation of mitochondria, is observed. The APP mouse models indicate impairment in anterograde transport while APP/PSEN1 and PSEN1 mouse models are characterized by dysfunction of anterograde and retrograde transport. Other mouse models (Tg2576 APP transgenic mice and APP/PS1) performed damaged, round, and swollen mitochondria coinciding with loss of oxidative activity and preceding the beginning of cognitive impairment and β-amyloid plaque creation. Both mitochondrial transportation and network impairment have a huge impact on synapse loss and, as a result, cognitive impairment [11]. Also *APOE* status may have an impact on mitochondrial function as non‐demented *APOE* ε4 individuals in comparison to non-*APOE* ε4 individuals have lower mitochondrial COX activity in the brain. It influences not only ETC but also glucose metabolism [14]. Alterations in genes related to mitochondrial energy metabolism and apoptosis were reported in young transgenic AD mice, which persisted throughout adulthood [15]. Early damage to genes involved in glucose metabolism and mitochondrial function, including AMP-activated protein kinase (AMPK), NRF1 and NRF2, and PGC-1α, as well as changes in oxidative phosphorylation complexes (OXPHOS) in APP/PS1 mice before atherosclerotic plaque, has been demonstrated [16]. All of this has the effect of decreasing mitochondrial activity and function. An increase in PGC-1α, which stimulates PPAR or sirtuin, reduces Aβ plaques so it has a neuroprotective effect in AD [17].

## 3. Oxidative stress AD

Oxidative stress is a condition in which the production of reactive oxygen species (ROS) exceeds the cell’s antioxidant defense system. It occurs through various pathways. Oxidative stress is associated with DNA defects, abnormal gene expression, defective enzyme activities, and energy failure. Reactive oxygen species (ROS) are by-products of metabolism. They are essential for the physiological functioning of the cells, but at high levels, they can be toxic [18]. Mitochondrial dysfunction, which causes increased production of reactive oxygen species (ROS), seems to contribute to the neurodegenerative mechanisms of Alzheimer’s disease. In this process, neurons are damaged and occur in various pathways. In AD, oxidative stress plays a very important role. In the cell, antioxidant enzymes such as superoxide dismutase (SOD), glutathione peroxidase (GPX), thioredoxins, glutaredoxins, and catalase, as well as non-enzymatic antioxidant factors such as vitamin E, vitamin A, vitamin C, uric acid, and carotenoids, perform the most important defense functions [19]. One of the first markers of AD is increased mtDNA oxidation. Having an age-related decline in mitochondrial function may be one of the first events in the pathogenesis of sporadic late-onset Alzheimer’s disease. According to the mitochondrial cascade hypothesis, age-related loss of mitochondrial function affects the expression and processing of APP, producing amyloid beta oligomers that accumulate in atherosclerotic plaques in Alzheimer’s disease [20]. The brain has a lot of readily oxidizable fatty acids and has a high demand for paramagnetic O2 and transition metal ions. In the oxidative production of ATP, the generation of ROS is inevitable and is a major cause of macromolecular damage (Table 1) [21]. ROS are typically reactive oxygen-containing molecules. The most common ROS is superoxide anion radical. It is mainly produced in complex I of the electron transport chain (ETC) in the inner mitochondrial membrane. The anions of this radical can move within 30 nm of formation; upon protonation, the superoxide becomes neutral HO2, which can cross the inner mitochondrial membrane and then accumulate in the cytoplasm [22]. The net effect of these ROS oxygen radicals is damage and death of vulnerable neurons in AD. Another ROS is H2O2. It diffuses easily across the inner mitochondrial membrane because it has a zero dipole moment, moving at least 1 µm from the site of production. H2O2 is less reactive than superoxide but can interact with reduced iron (Fe2 +), from iron-sulfur proteins or heme proteins, through Fenton chemistry, forming one of the most reactive ROS, free hydroxyl radicals (OH–) [23]. Hydroxyl radicals are the most reactive and have many possible interactions. They are formed at the metal site hence their higher site specificity. Disruption of metal metabolism has been observed in Alzheimer’s disease.

**Table 1:** Characteristics of the ROS

Reaction oxygen–containing molecules	Description
Superoxide anion radical	Move within 30 nm of formation; upon protonation, the superoxide becomes neutral HO_2_
Neutral HO_2_	Able to cross the inner mitochondrial membrane and then accumulate in the cytoplasm
H_2_O_2_	Diffuses easily across the inner mitochondrial membrane; it has a zero dipole moment; moving at least 1 µm; less reactive than superoxide
OH^–^ (free hydroxyl radicals)	Most reactive ROS; have wide range of possible interactions; they are formed of the metal site, hence their higher site specificity

## Glucose metabolism in AD

The human brain utilizes up to 25% of total body glucose which makes it one of the highest energy-consuming organs [7]. Multiple studies proved a connection between glucose metabolism impairment and the pathogenesis of Alzheimer’s disease. Impaired energy metabolism implicates mitochondrial dysfunction. Global reductions in glucose metabolism were detected by positron emission tomography (PET) with [18F]-fluoro-deoxyglucose (FDG) in AD brains 3 [10]. In the earliest stages of AD, decreased glucose metabolism predominates in posterior brain regions [24]. Glucose hypometabolism occurs decades before the onset of Alzheimer’s disease [7] and could be useful in the detection of AD at the early stages [24]. As glucose metabolism impairment and insulin resistance in AD brains are similar to abnormalities in type 2 diabetes, it is suggested that AD may be a brain representation of type 2 diabetes [25], and de la Monte et al. have named AD “type 3 diabetes” [10].

Glucose metabolism is a multi-stage process. It consists of glucose transportation and intracellular metabolism [7]. Transportation is provided by glucose transporters (GLUTs) [26]. Due to decreased GLUT1 and GLUT3 concentration in the AD patient’s brains, there is reduced glucose uptake. Aβ interferes with GLUT3 and impairs membrane translocation which is regulated by CKMP. Aβ inhibits CKMP which leads to glucose hypometabolism, higher glucose concentration in the brain, and is related to the severity of AD [10]. A decrease of GLUT4 in mouse models of AD has shown exacerbation of neurodegeneration and amyloid pathology and worsens cognitive function [10]. GLUT4 is insulin-regulated as insulin stimulates the expression of the GLUT4 gene and glucose transportation from the cytosol to the plasma membrane [27]. GLUT4 plays an important role in memory acquisition in the hippocampus; consequently, impairment in GLUT4 functioning may manifest as cognitive impairment [10].

In early AD stages, insulin levels are decreased, and as AD progresses insulin signaling and as a result glucose metabolism worsens [10], and insulin resistance increases. Insulin resistance reduces the level of insulin-degrading enzyme (IDE) [28] which metabolizes mitochondrial β-amyloids [10]. That leads to the conclusion that insulin signaling impairment disrupts mitochondrial Aβ removal. Mitochondrial Aβ might prompt mitochondrial dysfunction by Aβ-binding alcohol dehydrogenase (ABAD) which causes apoptosis and oxidative stress. In Du et al.’s study, the bound component of a transition pore–cyclophilin D also leads to oxidative stress and apoptosis and, as a study on AβPP mice showed, impacts on preservation of cognitive function [29].

## Mitochondria-targeted therapy for AD

Despite the significant development in medicine over recent years, no satisfying medication for AD is available. The current drugs only delay the progression of the disease. A better knowledge of particular molecular pathways, including mitochondrial dysfunction, may lead to novel therapeutic strategies for treatments. Table 2 summarizes the potential mitochondria-target therapy useful in AD.

**Table 2:** Mitochondria-targeted AD treatment

Potential drug	Mechanism of action	Results
Metformin	Inhibition of mitochondrial complex I	Activation of 5′-AMP-activated protein kinase (AMPK)
Capsaicin, coenzyme Q2, mucidin, and stigmatellin	Partial inhibition of mitochondrial complex I	Reduction of ROS level
MitoQ	Regulation of autophagy by induction of a pseudo mitochondrial membrane potential (PMMP)	Reduction of ROS level
BBB-penetrating and neuron-targeting peptides	Delivery antioxidants into neuronal mitochondria	Suppression of neuronal death and mitigating oxidative stress
Peptide SS-31	Inhibition cardiolipin peroxidation	Reduction of ROS level
SkQ	Direct neutralization of ROS due to the oxidation of plastoquinone, reduction of mitochondrial membrane potential	Reduction of ROS level, the hyperphosphorylation of amyloid-β1-42 (Abeta) and its precursor APP
MitoApocynin (MitoApo)	Inhibition NOX2 activation	Reduction of oxidative and nitrative stress, glial activation, and inflammatory reactions
MitoTEMPO	Increasing superoxide dismutase activity	Reduction of production of ROS and Aβ-induced lipid peroxidation
MitoVitE	Inhibition of lipid peroxidation	Reduction of peroxide-mediated oxidative stress, peroxide-induced caspase activation, and oxidative stress–induced cell death
Mitochondrial division inhibitor-1 (Mdivi-1)	Regulation of mitochondrial fusion	Reduction of production of ROS and lipid peroxidation
Ceramides	Induction Drp1 and activation caspases and inhibition of mitochondrial fission	Reduction of oxidative stress
SH-BC-893, *PAQ-22, DDQ*	Inhibition of mitochondrial fission	Reduction of oxidative stress
Beta-secretase (BACE 1) inhibitors	Reduction of mitochondrial membrane potential affects mitochondrial recycling and, as a result, upregulates cellular apoptotic signaling	Upregulation cellular apoptotic signaling

### Metformin

This well-known medicine is applied to many patients around the world. The data shows that DMT 2 is one of the risk factors for cognitive impairments, vascular dementia, and AD [30], one of the first therapeutic choices for diabetes mellitus type 2 (DMT2). It is also used to cure obesity, liver diseases, cardiovascular diseases, renal diseases, and even some types of cancer like breast cancer, endometrial cancer, or colorectal cancer [31]. Recent studies show its possible beneficial influence on mitochondrial dysfunctions. Metformin could inhibit mitochondrial complex I to result in defective cyclic AMP and protein kinase A signaling in response to glucagon and the stimulation of AMPK [32]. In some clinical trials, it was confirmed that taking metformin significantly improved the cognition of the patients compared to a placebo [33]. However, in the longitudinal study by Wu et al., there was no correlation between metformin treatment and longitudinal memory change found [34]. What is more, a case–control study by Imfeld et al. showed that long-term metformin uptake correlates with a slightly higher risk of developing AD [35]. This is why this topic must be further investigated.

### Partial mitochondrial complex I inhibition

Inhibition of the mitochondrial respiratory chain complex I, which contributes to creating reactive oxygen species (ROS), using small molecules, leads to a reduction in oxidative metabolism. There are various inhibitors of this part of the mitochondria membrane. They differ from each other according to the impact on ROS creation or the effect on the enzyme’s kinesthetics. Among those inhibitors that also prevent ROS, we may list the following substances such as capsaicin, coenzyme Q2, mucidin, and stigmatellin. Inhibitors that increase the level of ROS in the cell are piericidin A or rolliniastatin 1 and 2 [36]. What is more, there are some mutations in complex I that prevent NADH oxidation which at the same time stop ROS production [37]. Interestingly, one of the inhibitors of complex I, a small molecule called tricyclic pyrone compound (CP2), is presumed to be a perfect drug considering its safety profile and low toxicity and does not interfere with human receptors and ion channels [38]

CP2, even during stress conditions, reduced the proton leak. Many studies show that long molecule uptake resulted in considerable improvement of the nervous system homeostasis and the reduction of oxidative stress and inflammation which prevented neurodegeneration. At the same time, no signs of toxicity were observed [30]. Another advantage of CP2 is the fact that it prevents the formation of A*β* aggregates which prevents AD [39].

### The mitochondria-targeted antioxidant MitoQ

MitoQ is a mitochondria-targeted antioxidant with good neuroprotective features. By inducing a pseudo-mitochondrial membrane potential, it may affect mitochondrial respiration and cause autophagy. This process allows the cell to eliminate dysfunctional components [40]. MitoQ may be a renewable antioxidant [41]. The research on mice revealed that daily administration of this substance results in the improvement of the physical parameters and oxygen consumption [42]. Other studies show that daily addition of MitoQ to their drinking water prevented mice, which had mutant human transgenes responsible for AD early onset, from cognitive decline and AD-like pathologies [43]. However, MitoQ is not functioning when there is no coenzyme Q in mitochondria. This is because complex III cannot oxidize the reduced quinol form of MitoQ [44].

### New methods of drug administration to the neuronal mitochondria—biomimetic engineered nanosystems

Recent developments in biomimetic technology allow the development of new methods of treatment. The problems caused by the toxicity of synthetic materials were a serious complication in obtaining efficient transport to the neuronal mitochondria. In the past, there were used nanomaterials made of gold or silica, newly founded biomaterials such as human serum albumin red blood cell membrane–coated nanoparticle. It is modified to successfully reach the neuronal mitochondria. In the first studies, curcumin was used as a model drug [45].

Human serum albumin seems to be a perfect substance for medicine delivery due to its non-toxicity. The modified albumin could reach mitochondria in the nervous cell thanks to the positron emission tomography agent 7-(6-nitropyridin-3-yl) − 5H-pyrido[4,3-bindole (T807) which binds specifically to the nervous cells [46]. To reach the highest biocompatibility and to prevent interference with the RBC membranes, the lipid-insertion method was developed. The ligand may be thanks to this covering on the surface. The ligands after such a process are named with a special prefix such as DSPE-PEG3400-T807 [45].

### Antioxidant peptide SS-31

Antioxidant tetrapeptide SS-31 (D-Arg-Dmt-Lys-Phe-NH2; Dmt-2′,6′-dimethyl tyrosine) also known as MTP-131 decreases the impact of oxygen stress for the cell and stabilizes cardiolipin cytochrome c complex [47, 48]. In the research, it was revealed that administration of SS-31 to mice improves lipopolysaccharide (LPS)-induced memory, which is impaired due to oxidative stress. It also results in normal gene expression of antioxidant enzymes. The advantages of using SS-31 as a drug in neuro dysfunctional diseases may be also added to the fact of better administration of brain-derived neurotrophic factor (BDNF) signaling [49, 50].

### SkQ

It was the first mitochondria-targeted medicine used in clinical practice. It effectively eliminates ROS from the cells thanks to its structure—plastoquinone or thymoquinone which are antioxidants and responsible for electrophoretic transport of triphenylphosphonium—as an alternative rhodamine 19. They are bonded together by a linker of 10 carbon atoms [51].

Mitochondria-targeted antioxidants, such as SkQ, have a big advantage in that they may be used in very low concentrations which also limits the possibility of side effects on the organism. SkQ is a great hope for Alzheimer patients not only because of its limitation of ROS from the mitochondria. In the research, it was also confirmed that long-term administration of this drug declines the hyperphosphorylation of amyloid-β1-42 (Abeta) and its precursor APP [52]. However, the positive results were observed after a single administration of the molecule—the increase of the synaptic transmission was meaningful [51, 53].

### MitoApocynin (MitoApo)

MitoApo is a recently synthesized and orally administered derivative of apocynin. It targets mitochondria where it protects against oxidative damage and glial-mediated inflammation. Its efficiency in Parkinson’s disease (PD) was confirmed in numerous research; however, it is presumed to be also a possibility for treatment in other neurodegenerative diseases like AD. It must be taken under further investigation [54, 55].

### MitoTEMPO

It contains a free radical electron which is capable of eliminating mitochondrial superoxide. Its full name is 2,2,6,6-tetramethylpiperidin-1-yl)oxyl (TEMPO). This molecule highly improves the functioning of the mitochondrium—and limits the production of ROS and Aβ-induced lipid peroxidation. MitoTEMPO also mitigates Aβ-induced mitochondrial DNA (mtDNA) depletion. It was also confirmed that it limits the expression of mtDNA replication–related DNA polymerase gamma (DNA pol γ) [56, 57].

### MitoVitE

Mitochondrially targeted vitamin E reduces H2O2, inhibits caspase activation, and staves off apoptosis. Its full name is [2–3,4dihydro-6-hydroxy-2,5,7,8tetra-methyl-2H-1-benzopyran-2-yl], and this molecule is built of aromatic head with two ring structures and triphenylphosphonium bromide group (TPPB). MitoVitE is, due to TPPB, hydrophobic, which facilitates contact with mitochondrial membranes.

MitoVitE efficacy, even with a very small, nanomolecular portion during administration, is significantly high [10, 56].

## Mitochondrial division inhibitor-1 (Mdivi-1)

A new promising drug in fighting AD may be mitochondrial division inhibitor-1 (Mdivi-1). It is a dynamin-related protein 1 (Drp1)-specific inhibitor. Drp-1 is a mitochondrial protein responsible for membrane fragmentation [58, 59]. This small molecule has a big influence on mitochondria bioenergetics by influencing its dynamics, autophagy, ATP production, or the immune response. Mdivi-1 administration led to a reduction in H2O2 production and lipid peroxidation [57, 60].

In the research, it was found that Mdivi-1 improves mitochondrial function by limiting Aβ deposition [61]. What is more, it also positively influences mitochondrial fragmentation and deficit distribution [59].

### Ceramides

Ceramides are responsible for the regulation of many processes in the cells like proliferation or cellular aging. They are lipids built from sphingosine and a fatty acid [62]. The levels of ceramides in the serum or cerebrospinal fluid (CSF) are increased in patients with AD. They are responsible for the generation and aggregation of Aβ [63]. In the mitochondria, ceramides induced Drp1 and activated caspases. What is more, these lipids mediate the binding of Aβ to VDAC. Those processes led to neuronal cell death [64].

A new possibility of treatment would be to inhibit ceramides in Aβ-mediated toxicity in patients with AD. Research shows that the synthetic sphingolipid SH-BC-893 may be successfully used in rapid inhibition of ceramide-induced mitochondrial fission. What is more, it may lead to weight loss due to causing a similar effect as caloric restriction obtained in reducing food intake [65].

### Other inhibitors of mitochondrial fission

Mitochondrial fission is an important process in maintaining homeostasis, and it is a promising strategy for mitigating mitochondrial dysfunction. SH-BC-893 blocks palmitate, and ceramide-induced mitochondrial dysfunction prevents oxidative stress. Among other substances with these features, we may list 3-[2,6-diethylphenyl]quinazoline-2,4-dione (*PAQ-22*) [66], 1H-pyrrole-2-carboxamide [67], or *DDQ* (diethyl (3,4-dihydroxy phenethylamine) quinolin-4-yl]methylphosphonate) [68]. These molecules seem to be a new possibility of treatment for AD patients.

### Beta-secretase (BACE) inhibitors

Recently, there have been beta-secretase (BACE) inhibitors such as 5XFAD proposed. At present, their effectiveness was not confirmed in the research. However, this may be one of the brand-new investigation fields that may result in the future new treatment of AD [69].

### Lifestyle modifications

Many epidemiological studies suggest the beneficial impact of lifestyle modifications, including diet and physical activity. Physical activity improves the regulation of metabolic pathways, including insulin signaling, glucose, carbohydrates, and fatty acid metabolism. Exercises improve cognitive deficits in the APP/PS1 transgenic mouse. In the research, after 5 months of treadmill exercises, the level of APP phosphorylation and PS1 expression declined significantly [70]. Results of studies have shown that regular aerobic exercises have a positive influence on neurological skills. Among many improved areas, we may list concentration, learning capacity, and executive function. Regular activity also increased cortical thickness [71]. It has an anti-inflammatory effect and reduces the cardiovascular risk [72]. More frequent exercises stimulate the neurological system to restore thanks to enlarged levels of neurotrophins like brain-derived neurotrophic factors. They are secreted under the influence of ketone bodies, lactate, or muscle-derived myokines whose amount is increased after exercises [73]

Even though during exercises the level of oxidative stress is increased, regular physical exercises improve redox status thanks to vasodilatation and increasing the release of nitric oxide [74]. The level of the Aβ plaques in the patients with AD has also decreased thanks to the exercises [75], the same as the level of neuronal damage markers—neuron-specific enolase or catalase activity and ROS levels [10].

Thanks to the regular exercises, both mitochondrial biogenesis and regeneration [76], it also improves healthy aging and increases antioxidant capacities [77]. The beneficial influence of psychical effort in AD patients with mild variants of the disease preserved cognition [78, 79] awareness and problem-solving spheres of the memory score [80].

Research shows that also our dietary habits have a big influence on our neurological health. The Mediterranean diet is said to be balanced in the best possible way, providing the patient with the highest amount of nutrients and at the same time the lowest dose of saturated fats [81, 82]. Inherence of long-chain polyunsaturated fatty acids (PUFAs) in the daily diet is connected with a decreased risk of AD [83]. There were also benefits of a ketogenic diet on brain metabolism confirmed. It is explained by compensation for the AD brain glucose insufficient level [84]. Not only providing good products but also calorie restriction has a positive impact on cognition. Limitation of calories, however, with preservation of nutrients and avoidance of malnutrition, results in improving cognitive skills and slows down the aging process [85]. There are some substances like caffeine, curcumin, dapsone, metformin, resveratrol, or spermidine, which have similar effects as caloric restriction [86].

To sum up, exercise and diet may be a safe possibility for treatment, with many other advantages for the whole patient’s body, not only considering treating AD. Of course, it should be under the control of the patient’s doctor to adjust the intensity of the exercises and to choose an appropriately balanced diet.

## Summary

Alzheimer’s disease is the most common cause of dementia in the older population. The number of patients is predicted to significantly increase in the coming years. Currently, the therapy is limited to the symptomatic treatment. A better knowledge of pathogenesis has a crucial role in finding a novel therapy. The main aspect of this research was to focus on mitochondrial disorders leading to AD. Many studies show that oxidative stress is responsible for many neuronal damage as well as Aβ deposits. Modern therapy possibilities focus on decreasing ROS levels. The urgent concern is also developing new methods to transport drugs directly into the mitochondria, without causing any reactions with other body tissues. Much research is conducted by many scientists all over the world which gives hope for further investigation of this topic.

## Data Availability

All the data is available on the request of the authors.
